# Exploratory study: Evaluation of a symptom checker effectiveness for providing a diagnosis and evaluating the situation emergency compared to emergency physicians using simulated and standardized patients

**DOI:** 10.1371/journal.pone.0277568

**Published:** 2023-02-24

**Authors:** Laure Abensur Vuillaume, Julien Turpinier, Lauriane Cipolat, Arnaud-Dépil-Duval, Thomas Dumontier, Nicolas Peschanski, Yann Kieffer, Boris Barbat, Thomas Riquier, Vincent Dinot, Joris Galland

**Affiliations:** 1 Service d’Accueil des Urgences – SAMU 57, CHR Metz-Thionville, Metz, France; 2 IRL2958 Georgia Tech – CNRS, Metz, France; 3 Université de Lorraine, Vandoeuvre les Nancy, France; 4 Saint-Joseph Hospital, Paris, France; 5 Service d’Accueil des Urgences, CHU Rouen, Rouen, France; 6 Service d’Accueil des Urgences, CHU Rennes, Rennes, France; 7 Rennes University, Rennes, France; 8 Orsay Hospital, Groupe Hospitalier Nord Essonne, Paris, France; 9 Service d’Accueil des Urgences, APHP, Paris, France; 10 Plateforme Support au Développement Clinique des Solutions Numériques, Digital Medical Hub, Paris, France; 11 Plateforme d’appui à la Recherche Clinique, CHR Metz-Thionville, Metz, France; 12 Service de Médecine Interne, Hôpital Fleyriat, CH Bourg-en-Bresse, Viriat, France; Bilawal Medical College, Liaquat University of Medical and Health Sciences, PAKISTAN

## Abstract

**Background:**

The overloading of health care systems is an international problem. In this context, new tools such as symptom checker (SC) are emerging to improve patient orientation and triage. This SC should be rigorously evaluated and we can take a cue from the way we evaluate medical students, using objective structured clinical examinations (OSCE) with simulated patients.

**Objective:**

The main objective of this study was to evaluate the efficiency of a symptom checker versus emergency physicians using OSCEs as an assessment method.

**Methods:**

We explored a method to evaluate the ability to set a diagnosis and evaluate the emergency of a situation with simulation. A panel of medical experts wrote 220 simulated patients cases. Each situation was played twice by an actor trained to the role: once for the SC, then for an emergency physician. Like a teleconsultation, only the patient’s voice was accessible. We performed a prospective non-inferiority study. If primary analysis had failed to detect non-inferiority, we have planned a superiority analysis.

**Results:**

The SC established only 30% of the main diagnosis as the emergency physician found 81% of these. The emergency physician was also superior compared to the SC in the suggestion of secondary diagnosis (92% versus 52%). In the matter of patient triage (vital emergency or not), there is still a medical superiority (96% versus 71%). We prove a non-inferiority of the SC compared to the physician in terms of interviewing time.

**Conclusions and relevance:**

We should use simulated patients instead of clinical cases in order to evaluate the effectiveness of SCs.

## Introduction

Emergency department (ED) and primary care systems are overcrowded in France and abroad [1–6]. There seems to be a mismatch between upstream needs (ambulatory medicine) and the lack of downstream resources (number of emergency services, hospital beds) [7]. This pressure on the hospital is associated with increased morbidity and deterioration of care [8–10]. Several initiatives to reduce ED overcrowding emerged during the global COVID-19 pandemic through French government measures and the development of telemedicine [11]. However, these measures are currently insufficient to deal with the hospital crisis, particularly in France, which requires better regulation of patient flows, both upstream and downstream. As for the patients, they have more and more access to digital tools to help with diagnosis or orientation.

To partially meet this need, numerous symptom checkers (SC) are created [12, 13]. Questioning is an important phase of the clinical examination and plays an important role in medical reasoning and these tools propose to conduct this interrogation with a form or a chatbot [14]. They then offer several probable diagnoses for the patient and/or a referral. Since they can be a real help for the health system, it is important that SC can be rigorously evaluated. Several studies explored the accuracy of these tools. However, they often use a limited number of clinical cases or some especially created to evaluate this tool [15, 16]. These clinical cases may lack reproducibility and do not fully represent real life. This observation was made too in medical and paramedical education, and this is the reason that objective structured clinical examinations (OSCE) were created for since the 1970s [17]. OSCEs include “stations” which are basically clinical cases of simulated and standardized patients for fair assessment of clinical skills, including diagnostic skills [18]. There are only a few studies using OSCEs to evaluate teleconsulting, but current evidence points to the same reproducibility and effectiveness [18]. The exponential development of teleconsultations, already practiced by the regulation teams in the emergency call centers, show that the rapid questioning of the patient makes it possible to formulate several diagnostic hypotheses that are close to reality and adapted to the needs of patient care [19–21]. Thus, it is likely that simulated and standardized patients, such as those used in OSCEs, are suitable for the evaluation of SC and are closer to reality than the use of clinical cases.

The main objective of this study was to explore the effectiveness of a symptom checker against emergency physicians using simulated and standardized patients as an assessment method.

## Methods

To evaluate the practicability and the interest of using simulated and standardized patients, such as those used in OSCEs, to assess the diagnostic performance of an SC, we repeated the same assessment in front of a SC and in front of emergency physicians. The responses of the two study arms were then compared to the actual diagnosis of the simulated patient. Although it did not involve any real patient, we propose to report this study as a clinical study. We used the Standards for Strengthening the Reporting of Observational Studies in Epidemiology in simulation research (STROBE) reporting guideline to write our article [22].

### Creating simulated and standardized patients

Symptom checkers and doctors had to have access to the same level of information. We chose to give the doctor access only to the patient’s interrogation, without any possibility of clinical or complementary examination. Each simulated patient had to meet a certain number of quality criteria, including 1/ the concordance of the clinical history and symptoms with the main diagnosis 2/ the role is possible to perform in front of a software or in front of a doctor 3/ the patient does not present a state of vital distress, because the communication with him would be limited. In addition, the simulated and standardized patient histories includes standard questions and answers. If an information was not given, the patient was asked to answer "I don’t know." Finally, the actors were trained to the role of standardized patient by following the recommendations issued in the OSCEs organization.

#### Choice of the nosological frameworks

We raised an expert panel composed of general practitioners, emergency physicians and internists to define the most common diseases encountered during unscheduled care consultations. The expert team selected 44 diseases. The diseases coded by the symptom checker were not known by the expert panel to allow an objective evaluation that could be similar to real life.

#### Writing and verification of simulated patients’ charts

The expert college developed a consensual template for the simulated patient form. In order to ensure reproducibility, a test of this form was carried out on the diagnosis of myocardial infarction by each doctor who wrote it. This permitted to correct the way it was written if necessary, and standardized it. Each expert wrote 1 clinical case per pathology. Each form was independently reviewed by two doctorss to ensure that the quality criteria (see above) were fulfilled and to obtain a consensus.

### Study design

**Fig 1:**
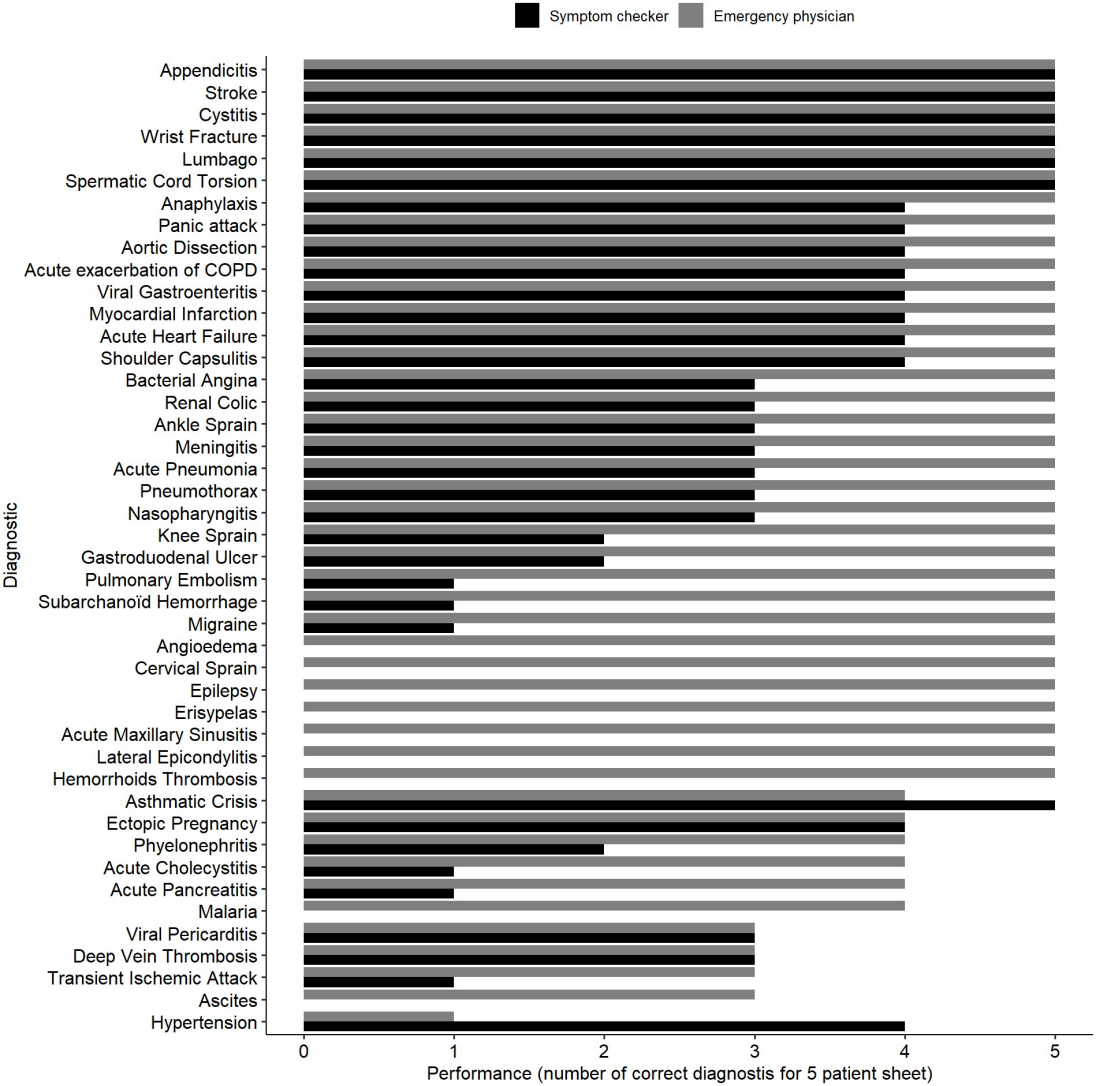


We performed a prospective randomized, non-inferiority study with simulated patients. The gold standard was the diagnosis of each simulated patient (Fig 1).

#### Assumptions and judgment criteria

The study hypothesis was that the effectiveness of a symptom checker was not inferior to an emergency physician. The expected performance of an expert emergency physician was 90% success on the primary endpoint and 80% success on the secondary endpoint.

The primary endpoint was the percentage of correct answers on the main diagnosis versus gold standard. The secondary endpoints were the percentage of correct answers on the main and secondary diagnoses versus the gold standard, the duration of the interview, the number of questions and the content of the questions to reach an identical result.

#### Justification of the number of subjects needed

We expect 90% of correct answers for the emergency physician, and probably about the same for the expert system, the discordant pairs will be rare. We can retain 5% for the two types of discordance. For the primary analysis, with a 15% of non-inferiority margin, a sample size of 205 patient sheets would achieve at least 90% power to detect non-inferiority at a one-sided alpha of 2.5%.

#### Device

The SC that allowed us the evaluation is based on a neural network technology. We obtained the agreement of the software to carry out our exploratory study.

### Course of the study

A total of 220 clinical cases were written by the expert panel of physicians. Each standardized situation was played twice by an actor previously trained for the role: once in front of the symptom checker, once in front of an experienced emergency physician. In the different groups of the study, we collected the order of the questions asked and we measured the time of the interrogation. Interviews in front of experienced physicians were conducted by conference calls between September 2021 and November 2021 with the emergency physician, the actor, and two study evaluators. The order of clinical cases was randomly assigned to 2 study groups by computerized randomization. Symptom checker face-to-face interviews were conducted between September 2021 and November 2021. Situations that did not lead to a diagnosis were repeated twice to ensure the result and the absence of a technical problem. At the end of the evaluations, a qualitative analysis of the physicians’ diagnostic and the symptom checker mistakes (severity and probable reason for the mistake) was performed consensually by 2 physicians.

Finally, the evaluating doctors were asked to fill a satisfaction questionnaire about their experience with the study (Likert scale).

### Statistical analysis

All data were reported the following manner: medians (Q1,Q3) for quantitative variables and numbers (percentages) for qualitative variables. Mcnemar test and paired Wilcoxon non-parametric test were used to compare qualitative and quantitative variables, respectively.

If primary analysis failed to detect non-inferiority, we had planned a superiority analysis to understand if non-inferiority is not reached because doctors have a better diagnosis performance than software.

### Ethics

This simulation study did not require the regulatory framework of research involving the human person, requiring authorization from an ethics committee. Our study was not performed on real patients but on simulated patients (actors). The scope of the research was outside of clinical research and did not require consent. All the people involved in this research, actors and medical evaluators, are associated with the publication. The opinion of the ethics committee was not necessary.

## Results

### Simulated patients

The characteristics of the simulated patients are described on Table 1.

**Table 1:** Characteristics of simulated patients.

General characteristics	N = 220^1^ (%)		N = 220^1^ (%)
age	35 (25, 56)		
gender			
female	96 (44%)		
male	124 (56%)		
**Diagnostics**			
Transient Ischemic Attack	5 (2%)	Ectopic Pregnancy	5 (2%)
Anaphylaxis	5 (2%)	Subarchanoïd Hemorrhage	5 (2%)
Bacterial Angina	5 (2%)	Hypertension	5 (2%)
Angioedema	5 (2%)	Myocardial Infarction	5 (2%)
Appendicitis	5 (2%)	Lumbago	5 (2%)
Ascites	5 (2%)	Meningitis	5 (2%)
Stroke	5 (2%)	Migraine	5 (2%)
Acute Cholecystitis	5 (2%)	Malaria	5 (2%)
Renal colic	5 (2%)	Acute Pancreatitis	5 (2%)
Panic Attack	5 (2%)	Viral Pericarditis	5 (2%)
Asthmatic Crisis	5 (2%)	Acute Pneumonia	5 (2%)
Cystitis	5 (2%)	Pneumothorax	5 (2%)
Aortic dissection	5 (2%)	Acute Heart Failure	5 (2%)
Pulmonary embolism	5 (2%)	Pyelonephritis	5 (2%)
Cervical Sprain	5 (2%)	Nasopharyngitis	5 (2%)
Ankle Sprain	5 (2%)	Acute Maxillary Sinusitis	5 (2%)
Knee Sprain	5 (2%)	Lateral Epicondylitis	5 (2%)
Epilepsy	5 (2%)	Shoulder Capsulitis	5 (2%)
Erysipelas	5 (2%)	Hemorrhoids Thrombosis	5 (2%)
Acute exacerbation of COPD	5 (2%)	Spermatic Cord Torsion	5 (2%)
Wrist Fracture	5 (2%)	Deep Vein Thrombosis	5 (2%)
Viral Gastroenteritis	5 (2%)	Gastroduodenal Ulcer	5 (2%)
		^*1*^ Median (IQR); n (%)	

COPD = chronic obstructive post-smoking bronchitis

At the end of the evaluation period, the list of diagnoses was compared with the list of diagnoses in the SC. There were 4 different diagnoses, or 20 patients, not known by the tool (Table 2).

**Table 2:** Description of unknown diagnoses by the symptom checker.

	N = 20 (%)
Angioedema	5 (25%)
Ascite	5 (25%)
Cervical Sprain	5 (25%)
Hemorrhoidal thrombosis	5 (25%)

### Non-inferiority and superiority analyses

The symptom checker is not inferior in terms of interviewing time.

The emergency physician was superior to SC in terms of principal diagnosis (81% versus 30%) and association of principal and secondary diagnosis (92% versus 52%). In terms of patient triage (vital emergency or not), there is still a medical superiority (96% versus 71%). The overall results are presented in Table 3.

**Table 3:** Non-inferiority and superiority analysis.

Characteristic	Symptom Checker, N = 220	Emergency Physicians N = 220	Non inferiority	Superiority
Principal diagnosis	67 (30%)	178 (81%)	p = 1	p = 0.02*
Principal or secondary diagnosis	114 (52%)	202 (92%)	p = 1	p = <0.001*
Time (minutes Q1-Q3)	2 (1, 3)	2 (2, 3)	p = <0.001*	
Triage	156 (71%)	211 (96%)	p = 1	p = <0.001*

### Evaluation of diagnostic discordance

We analyzed diagnostic discordance between the SC and the emergency physician. SC had a better diagnostic performance on the following pathologies: cystitis, acute viral pericarditis, asthma attack, and arterial hypertension.

**Fig 2:**
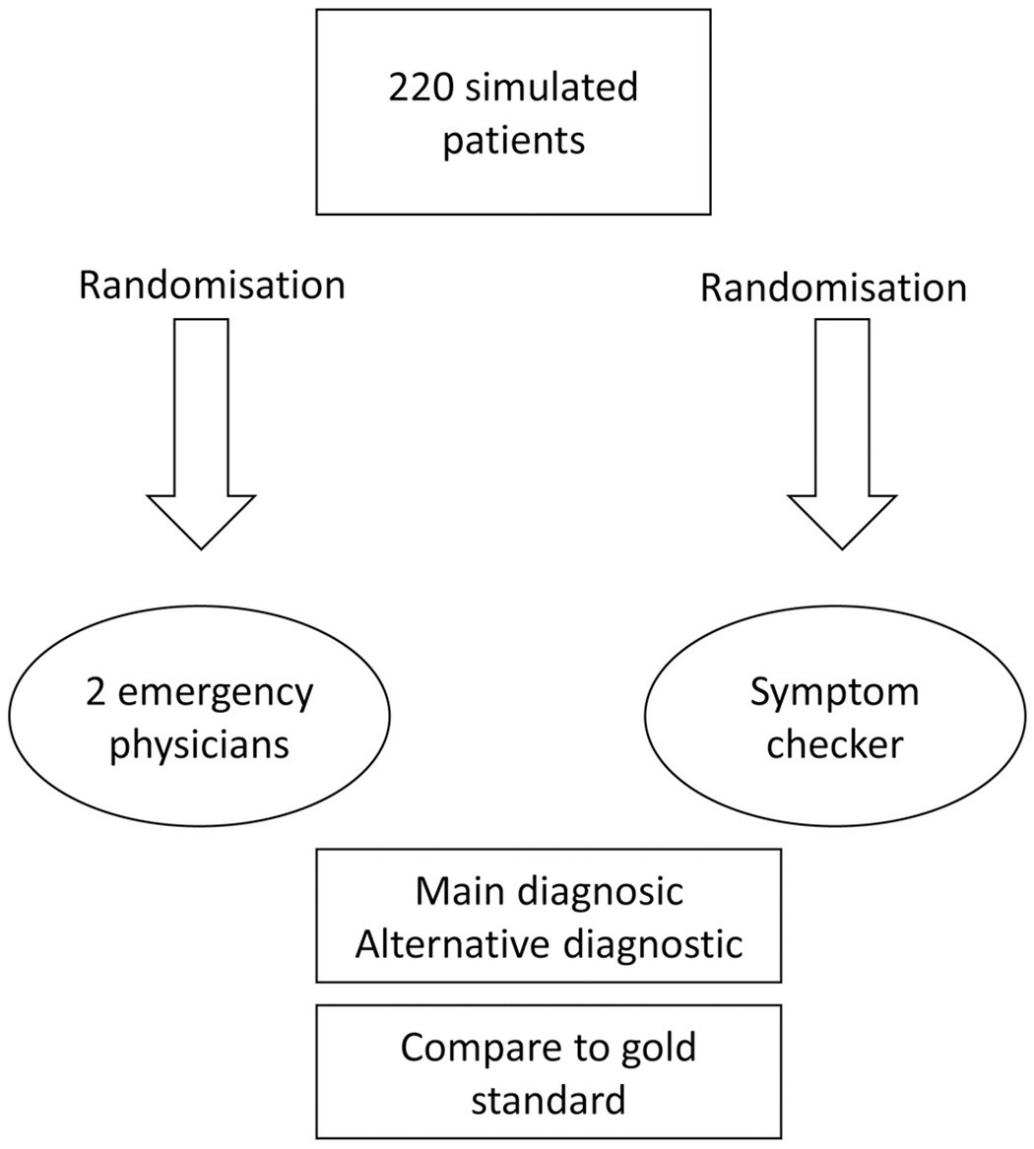


The overall diagnostic performance for each disease is reported in Fig 2.

### Evaluation by evaluator physicians

Both physician evaluators were asked to fill a questionnaire about their experience and rated on a Likert scale. They both strongly agreed with the statement “The clinical situations presented by the simulated patients fell within the definition of unscheduled care* (*actual or felt emergency)” and were “mostly agree” and “strongly agree” with the statement “The clinical situations presented by the simulated patients were similar to real life situations”. Finally, when confronted with the statement “the acting allowed me to feel in my daily practice”, both physicians agreed.

## Discussion

We have, for the first time to our knowledge, evaluated a SC with simulated and standardized patients as used in OSCEs. The medical history alone appears to be sufficient to make a reasonable diagnostic hypothesis. It seems that OSCEs can be widely adapted in the field of training and evaluation of teleconsultations, telephone consultations, or medical regulation. Specifically regarding the evaluation of SC, it is important to know that there is no reference method [16, 23, 24]. We have objectively evaluated a SC software, in an independent way and without knowing its capabilities. In our opinion, this study was conducted in an approach closer to real life than that proposed in clinical cases. and could be applied in software evaluation and also for educational purposes.

The new computer tools such as SC or the wider access to remote consultations have strong advantages with interesting perspectives in terms of public health. From an organizational point of view, these tools can also be interesting for preparing a consultation prior to receive the patient They allow the referencing and standardization of patient complaints in an automated digital format that can be adapted in any language. Faced with a growing request for care and an increase in the difficulty for health professionals linked in part to the COVID-19 pandemic, the use of assistance without human cost would be relevant [25]. Some countries, such as Sweden, have already developed innovative tools to release the healthcare system, such as the SC system, which allows for efficient dispatch of paramedics during working hours, and telephone dispatch during on-call hours [26–28]. Like a gatekeeper, and rather than trying to reason like a physician to give a diagnosis to the patient, the SC would allow the patient to be directed to the right level of care (home, ambulatory, hospital). It seems very likely that there would be good patient compliance with the use of this type of tool [29]. However, there are still too many obstacles to the use of both HC software by health professionals and the performance of remote consultations, and these need to be overcome by 1/ increasing their level of effectiveness, 2/ integrating them into the overall care process, and 3/ increasing the quality of studies and training on them [30].

However, to this day, the skills of a symptoms checker are still much lower than those of a doctor, which suggests that the profession has a long way to go. Several elements can explain these differences. On the one hand, it seems that most SC know only a limited number of diagnoses compared to doctors, which was the case in our study. On the other hand, SC are most often constructed from patients’ clinical cases. These cases may reflect a single physician experience and not a consensus or reality. Some SC do not take into account the patient’s history or current treatments, which alone can guide the diagnosis [31]. Finally, the medical diagnostic process involves complex mechanisms, depending on the experience and the medical specialty [32]. These elements are important, especially concerning emergency medicine [33]. SC should probably learn more about diagnostic reasoning to improve their performance, as diagnostic reasoning is a key part of learning to be a physician [34]. Evidence-based medicine and rigorous clinical evaluation seem necessary, with OSCEs appearing to be a promising lead [25].

### Limitations

Our study has several limitations. First, this is an exploratory study. Indeed, we wanted to observe if the method we propose could be relevant for the evaluation of a SC. The reality of the simulated patients and clinical situations was recognized as similar to reality by the two evaluating physicians. Secondly, 5 diagnoses were not yet known by the evaluated SC, which probably underestimates its performance. But this approach assumed by the expert committee allowed a “real life” approach. Furthermore, we only evaluated one software that uses a neural network technology. It would be particularly interesting to explore, through our proposed method, other software technologies. Finally, from an educational point of view, an in-depth study of the formative aspect of remote OSCEs must be carried out to confirm these results.

## Conclusions

Through this exploratory study, we propose to apply simulated and standardized patients as used in OSCEs to evaluate the diagnostic performance of SC and physicians in situations where only the patient’s voice is accessible (telephone consultation, medical regulation). This type of evaluation should be extended to other types of software in order to provide scientific evidence of the application of tools used in pedagogy to clinical research, but also to deepen the evaluation for educational purposes.
